# Síndrome Hiperinflamatória como Mecanismo de Lesão Cardíaca

**DOI:** 10.36660/abc.20210146

**Published:** 2021-03-03

**Affiliations:** 1 Universidade de São Paulo Faculdade de Medicina Hospital das Clínicas São PauloSP Brasil Instituto do Coração do Hospital das Clínicas da Faculdade de Medicina da Universidade de São Paulo, São Paulo, SP – Brasil

**Keywords:** Hemofagocítico, Insuficiência Cardíaca, NT-proBNP, Hiperinflamação

A síndrome hemofagocítica (SHF) ou linfohistiocitose hemofagocítica (LHH) é uma doença inflamatória sistêmica aguda e rapidamente progressiva caracterizada por citopenia, produção excessiva de citocinas e hiperferritinemia. As manifestações clínicas comuns da LHH são febre aguda contínua, linfadenopatia, hepatoesplenomegalia e falência de múltiplos órgãos. Consiste em duas manifestações distintas que podem ser difíceis de distinguir: uma forma primária é vista principalmente em crianças e é causada por diversas mutações com herança genética e sua forma secundária pode ser maligna, infecciosa ou autoimune/autoinflamatória sem um gatilho genético subjacente identificável.^1^^,^^2^

O estado imune hiperinflamatório é causado pela ausência de infrarregulação normal por macrófagos e linfócitos ativados. A maioria dos pacientes com LHH apresenta comprometimento da função citotóxica de células exterminadoras naturais ou células NK (natural killer cells) e linfócitos citotóxicos (LCTs), juntamente com ativação excessiva de macrófagos.^3^ A falta de regulação por feedback resulta em atividade excessiva dos macrófagos e altos níveis de interferon gama e outras citocinas. A produção excessiva de citocinas por macrófagos, células NK e LCTs é um mediador primário de dano tecidual.^4^

Pode-se suspeitar da LHH em bebês, crianças ou adultos com citopenia de causa desconhecida, hepatite ou achados inflamatórios do sistema nervoso central. Os pacientes apresentam febre de causa desconhecida, hepatoesplenomegalia, histórico de episódios semelhantes a LHH, histórico familiar de LHH ou doença genética conhecida associada a LHH.^5^ O desfecho da síndrome de LHH não tratada costuma ser fatal. A complexidade clínica, a raridade e a diversidade das causas exigem que os médicos prestem muita atenção a essa possibilidade diagnóstica. O reconhecimento imediato da síndrome de LHH e o diagnóstico das causas subjacentes da LHH são vitais para permitir o tratamento imediato e apropriado.^2^

Nesta edição dos Arquivos Brasileiros de Cardiologia, os autores avaliaram dados retrospectivos de 39 pacientes com diagnóstico de LHH secundária de acordo com os critérios diagnósticos da LHH de 2004.^6^ Eles mostraram que marcadores laboratoriais não tradicionais de inflamação aumentada estão significativamente associados à mortalidade dos pacientes durante o seguimento. Entre os marcadores, tanto o valor final da porção N-terminal do pró-hormônio do peptídeo natriurético tipo B (NT-proBNP) quanto os valores da variação dos parâmetros laboratoriais durante a internação apresentaram maior mortalidade. Esse marcador é útil para predizer mortalidade por insuficiência cardíaca com fração de ejeção preservada e reduzida. Portanto, este estudo mostra a relação entre o NT-proBNP e a sobrevida do paciente durante a hospitalização.^6^

O NT-proBNP está associado a dano miocárdico, estresse, fibrose e inflamação sistêmica. Níveis elevados de NT-proBNP constituem um critério diagnóstico independente para insuficiência cardíaca com fração de ejeção preservada.^7^^,^^8^ Sabe-se que estados inflamatórios podem desencadear lesões cardíacas, como a insuficiência cardíaca, descrita nos casos de COVID-19 e também em outras doenças com alto índice de inflamação.^9^ No entanto, é importante entender que níveis moderadamente altos de NT-proBNP em pacientes com inflamação devem ser interpretados com cautela: tal estado inflamatório pode elevar a razão NT-proBNP/BNP e superestimar a gravidade da IC. A inflamação pode aumentar o clearance mediada por protease ou receptor de BNP e NT-proBNP, que não é afetado por esses mecanismos de clearance, resultando em um aumento da razão NT-proBNP/BNP.^10^

Em outros acometimentos que não a LHH, níveis elevados de NT-proBNP estão correlacionados com desfechos desfavoráveis. Uma revisão sistemática de 46 estudos de choque séptico concluiu que níveis elevados de BNP e NT-proBNP estiveram associados a aumento da mortalidade, independentemente da disfunção cardíaca. Além disso, esse marcador pode ser usado para distinguir entre choque séptico e cardiogênico.^11^ Em outro estudo com pacientes diagnosticados com mieloma múltiplo (MM), os níveis de NT-proBNP aumentaram significativamente, assim como outros marcadores de gravidade da doença. O MM pode causar lesão cardíaca como amiloidose cardíaca, mas, em alguns casos, o NT-proBNP sugere inflamação como a ligação entre o MM e o comprometimento cardíaco funcional.^12^

Conforme apresentado neste editorial, o NT-proBNP é um marcador de disfunção cardíaca, mas também está associado a condições inflamatórias graves. Essa correlação pode ajudar os médicos a identificar altos estados inflamatórios a fim de introduzir tratamento imediato. Além disso, se o paciente apresentar disfunção cardíaca, o marcador desempenha um papel importante no diagnóstico dessa condição, possibilitando prevenir danos ao coração como hipervolemia ou o uso de medicamentos depressores cardíacos.^6^
