# Supplementary material for: Síndrome Hiperinflamatória como Mecanismo de Lesão Cardíaca
Source: Arq Bras Cardiol. 2021 Mar 3;116(3):402–3. [Article in Portuguese] doi: 10.36660/abc.20210146 (PMC8159557; doi:10.36660/abc.20210146)
Supplement: Translation

# Translation: Hyperinflammatory Syndrome as a Cardiac Injury Mechanism

**Affiliations:** 1 São PauloSP Brazil Instituto do Coração do Hospital das Clínicas da Faculdade de Medicina da Universidade de São Paulo, São Paulo, SP – Brazil

**Keywords:** Hemophagocytic, Heart Failure, NT-proBNP, Hyperinflammation

Hemophagocytic syndrome (HPS) or hemophagocytic lymphohistiocytosis (HLH) is an acute and rapidly progressive systemic inflammatory disorder characterized by cytopenia, excessive cytokine production and hyperferritinemia. Common clinical manifestations of HLH are acute unremitting fever, lymphadenopathy, hepatosplenomegaly and multiple-organ failure. It consists of two different conditions that may be difficult to distinguish: a primary form is mostly seen in children and is caused by various mutations with genetic inheritance and a secondary HLH may be secondary to a malignant, infectious, or autoimmune/autoinflammatory stimulus without an identifiable underlying genetic trigger.^1^^,^^2^

The hyperinflammatory immune state is caused by the absence of normal downregulation by activated macrophages and lymphocytes. Most patients with HLH have impaired cytotoxic function of natural killer (NK) cells and cytotoxic lymphocytes (CTLs), coupled with excessive activation of macrophages.^3^ Lack of feedback regulation results in excessive macrophage activity and high levels of interferon gamma and other cytokines. Excessive cytokine production by macrophages, NK cells, and CTLs is a primary mediator of tissue damage.^4^

HLH may be suspected in infants, children or adults with unknown cytopenia, hepatitis or inflammatory central nervous system findings. Patients present unknown fever, hepatosplenomegaly, prior HLH-like episodes, family history of HLH or a known genetic disorder associated with HLH.^5^ The outcome of untreated HLH syndrome is often fatal. Clinical complexity, rarity, and diversity of causes require the physicians pay a great deal of attention to this diagnostic possibility. Prompt recognition of the HLH syndrome and diagnosis of the underlying causes of HLH is vital to enable urgent and appropriate treatment.^2^

In this edition of Arquivos Brasileiros de Cardiologia, the authors evaluated retrospective data of 39 patients with secondary HLH diagnosis according to HLH 2004 diagnostic criteria.^6^ They showed that non-traditional laboratory markers of increased inflammation are significantly associated with patient mortality during follow-up. Among the markers, both the N-terminal prohormone of brain natriuretic peptide (NT-proBNP) final value and laboratory parameter variation values during hospital stay presented a higher mortality rate. This marker is useful to predict mortality regarding heart failure both with preserved and reduced ejection fraction. Therefore, this study shows the relationship between NT-proBNP and patient survival during hospitalization.^6^

NT-proBNP is associated with myocardial damage, stress, fibrosis and systemic inflammation, and high NT-proBNP levels constitute an independent diagnostic criterion for heart failure with preserved ejection fraction.^7^^,^^8^ It is known that inflammatory states may trigger cardiac injury such as heart failure, which was described in COVID-19 diseases as well as others with high inflammation rate.^9^ However, it is important to understand that a moderately high NT-proBNP level in a patient with inflammation must be interpreted with caution: such inflammatory state may increase NT-proBNP/BNP ratio and overestimate HF severity. Inflammation may increase protease or receptor-mediated clearance of BNP and NT-proBNP, which is not affected by these clearance mechanisms, resulting in an increased NT-proBNP/BNP ratio.^10^

In conditions other than HLH, high NT-proBNP levels are correlated with unfavorable outcomes. A systematic review of 46 studies of septic shock concluded that high levels of BNP and NT-proBNP were associated with increased mortality regardless of cardiac dysfunction. Furthermore, this marker may be used to distinguish between septic and cardiogenic shock.^11^ In another study with patients diagnosed with multiple myeloma (MM), NT-proBNP levels rose significantly such as other disease severity markers. MM may cause cardiac injury such as cardiac amyloidosis, but, in some cases, NT-proBNP suggests inflammation as the link between MM and functional cardiac impairment.^12^

As presented in this editorial, NT-proBNP is a marker of cardiac dysfunction, but it is also associated with severe inflammatory conditions. This correlation can help physicians identify high inflammatory states in order to introduce prompt treatment. Furthermore, if the patient presents cardiac dysfunction, the marker plays an important role in find out such condition. This makes it possible to prevent harm against the heart such as hypervolemia or the use of cardiac depressants drugs.^6^
